# Icotinib With Concurrent Radiotherapy vs Radiotherapy Alone in Older Adults With Unresectable Esophageal Squamous Cell Carcinoma: A Phase II Randomized Clinical Trial

**DOI:** 10.1001/jamanetworkopen.2020.19440

**Published:** 2020-10-07

**Authors:** Honglei Luo, Wei Jiang, Li Ma, Peng Chen, Min Fang, Lingyu Ding, Yuhui Hua, Dexi Du, Zhao Jing, Ruifei Xie, Yaqi Song, Jiayang Wang, Rongjing Zhou, Zhifeng Tian, Shixiu Wu

**Affiliations:** 1 Department of Radiation Oncology, the Affiliated Huaian No.1 People’s Hospital of Nanjing Medical University, Huaian, China; 2 Department of Radiation Oncology, National Cancer Center/National Clinical Research Center for Cancer/Cancer Hospital & Shenzhen Hospital, Chinese Academy of Medical Sciences and Peking Union Medical College, Shenzhen, China; 3 Department of Radiation Oncology, Zhejiang Provincial People’s Hospital, People’s Hospital of Hangzhou Medical College, Hangzhou, Zhejiang, China; 4 Department of Medical Oncology, Hangzhou Cancer Hospital, Hangzhou, China; 5 Department of Pharmacy, Hangzhou Cancer Hospital, Hangzhou, China; 6 Department of Radiation Oncology, Lishui Central Hospital, Lishui, China; 7 Department of Radiation Oncology, Hangzhou Cancer Hospital, Hangzhou, China; 8 Department of Bio-informatics, Hangzhou Cancer Hospital, Hangzhou, China; 9 Department of Pathology, Hangzhou Cancer Hospital, Hangzhou, China

## Abstract

**Importance:**

Palliative radiotherapy (RT) is generally recommended for older patients with esophageal squamous cell carcinoma (ESCC) with poor prognosis. A new combination treatment is therefore needed.

**Objective:**

To assess the efficacy and toxicity of RT plus icotinib vs RT alone in older patients with ESCC.

**Design, Setting, and Participants:**

This randomized, multicenter, open-label, phase II clinical trial was conducted in China, with enrollment between January 1, 2015, and October 31, 2016. Patients aged 70 years or older with clinical stage T2 to T4, N0/1, M0/1a unresectable (because of comorbidities, T4 disease, unresectable lymph node, or refused surgery) ESCC were randomized 1:1 to receive RT plus icotinib or RT alone. Radiation was prescribed at 60 Gy in 30 fractions in both groups, and icotinib was administered at a dosage of 125 mg 3 times a day in the RT plus icotinib group. The last follow-up was completed on June 30, 2019, and data were analyzed from July 1 to September 30, 2019.

**Interventions:**

Patients were randomized to either RT plus icotinib or RT alone.

**Main Outcomes and Measures:**

The primary end point was overall survival (OS). Treatment-related toxic effects were evaluated. Immunohistochemistry was performed to analyze epidermal growth factor receptor (EGFR) expression if available.

**Results:**

A total of 127 patients (median age, 76 years [range, 70-91 years]; 76 men [59.8%]) were enrolled and were eligible for survival analysis. Median OS was 24.0 (95% CI, 22.2-25.8) months in the RT plus icotinib group vs 16.3 (95% CI, 13.8-18.8) months in the RT group (hazard ratio, 0.53; 95% CI, 0.33-0.87; *P* = .008). No difference was observed in grades 3 or 4 adverse events. Patients with EGFR overexpression had a significantly better median overall survival (not reached vs 16.3 months [range, 2.6-45.1 months]; *P* = .03) in the RT plus icotinib group.

**Conclusions and Relevance:**

In this randomized clinical trial, icotinib plus RT was well tolerated and improved OS in older patients with ESCC relative to RT alone. Patients with EGFR overexpression benefitted more from icotinib with RT.

**Trial Registration:**

ClinicalTrials.gov Identifier: NCT02375581

## Key Points

**Question:**

Is icotinib plus radiotherapy superior to radiotherapy alone in patients with esophageal squamous cell carcinoma?

**Findings:**

In this randomized clinical trial that included 127 patients 70 years or older with esophageal squamous cell carcinoma, patients treated with icotinib plus radiotherapy had a median overall survival of 24.0 months, whereas those treated with radiotherapy alone had a median overall survival of 16.3 months.

**Meaning:**

In this study, icotinib plus radiotherapy was tolerable and improved overall survival in older patients with esophageal squamous cell carcinoma.

## Teaser

This randomized clinical trial assesses the efficacy and toxicity of radiotherapy plus icotinib compared with radiotherapy alone in older patients with unresectable esophageal squamous cell carcinoma (ESCC) in China.

## Introduction

Concurrent chemoradiation therapy (CCRT) remains the standard therapy for unresectable esophageal squamous cell carcinoma (ESCC).^1,2^ However, its feasibility and efficiency for older patients are being challenged.^3,4,5^ Serious acute toxic effects, such as grade 3/4 hematologic toxicity, esophagitis, and pneumonitis, were commonly observed.^4,6,7^ In a retrospective analysis, only 33.3% of older patients completed chemoradiation, whereas 68.3% of nonelderly patients completed it.^4^ Treatment-related death was suspected in up to 18% of patients older than 75 years who underwent CCRT, even when both chemotherapy and radiotherapy (RT) were reduced in dose and field when necessary.^8^ Concurrent chemoradiation therapy showed no superiority over RT alone in survival.^6^ Palliative RT is generally recommended for older patients, but with limited survival benefit.^1,9^

Epidermal growth factor receptor (EGFR) is overexpressed in 30% to 70% of patients with ESCC and is associated with poor prognosis and an inferior response to conventional treatment.^10^ Several phase III studies have tried unsuccessfully to combine EGFR monoclonal antibody (cetuximab) with chemoradiation in esophageal cancer.^11,12,13^ An impaired overall survival (OS) was reported in the SCOPE1 trial,^12^ which may be partly due to the additional toxic effects. Thus, tolerance and toxicity should be carefully evaluated when delivering combined therapy, especially in older patients. The EGFR tyrosine kinase inhibitor (TKI) helps disrupt cell growth pathways and makes cells more sensitive to RT.^14,15^ Icotinib, an oral EGFR TKI, has been reported to markedly inhibit the proliferation of the human epidermoid squamous carcinoma A431 cell line with a high level of EGFR.^16^ Wang et al^17^ have evaluated the feasibility of icotinib in patients with advanced ESCC with EGFR overexpression. The efficacy and safety of combined EGFR inhibitors and RT has also been confirmed in patients with ESCC who are intolerant of CCRT.^18,19^ The pilot study of erlotinib plus RT revealed a 2-year survival rate of 44.4% with a reasonable safety profile.^18^

We therefore launched a randomized, multicenter, open-label, phase II clinical trial to investigate whether icotinib in combination with concurrent RT was superior to RT alone in older patients with ESCC.

## Methods

### Study Design

This randomized, multicenter, open-label, phase II clinical trial was conducted at Hangzhou Cancer Hospital, Huaian First People's Hospital and Lishui Central Hospital in China. The trial protocol (Supplement 1) was approved by the institutional review board and independent ethics committee of each participating center. All patients provided written informed consent. Patients’ records were anonymized and deidentified prior to analysis. This study followed the Consolidated Standards of Reporting Trials (CONSORT) reporting guideline.

### Patient Eligibility

Eligible patients had histologically confirmed unresectable or medically inoperable ESCC clinically staged as T2 to 24, N0/1, M0/1a according to the 2002 International Union against Cancer TNM staging system and unsuitable for concurrent chemoradiation owing to comorbidities or patient choice.

Other eligibility criteria included age of 70 years or older; Eastern Cooperative Oncology Group (ECOG) performance status of 2 or less; adequate renal, liver, and bone marrow reserve; and adequate nutritional intake.

Ineligibility criteria included history of other tumors, tracheoesophageal fistula, metastatic disease, severe dysphagia causing an inability to swallow icotinib, prior systemic treatment or thoracic radiation, and significant medical or psychiatric illnesses that would compromise the proposed treatment.

### Randomization and Masking

Eligible patients were randomly assigned in a 1:1 ratio to receive RT plus icotinib or RT alone and stratified by T stage (T2 vs T3 vs T4), N stage (N0 vs N+), and ECOG performance status (0 or 1 vs 2). The minimization method was used to ensure balanced stratification.^20^ Central randomization was done with the use of the random number table method. Patients, study staff, and the sponsor were unmasked to treatment assignment.

### Baseline Characteristics Evaluation

Age, sex, ECOG performance score, tumor stage, location, length, weight loss, and dysphagia were assessed. Degree of dysphagia was evaluated using the Atkinson scale (scores range from 0 to 4, with higher scores indicating greater dysphagia).^21^

### Treatment Schedule

Radiotherapy was administered in both groups using high-energy linear accelerators with conventional fraction, 3-dimensional conformal technique or intensity-modulated RT. Gross tumor volume was defined as the primary tumor and any enlarged regional lymph nodes. Clinical target volume included the gross tumor volume plus a 5-cm expansion superiorly and inferiorly along the length of the esophagus and a 1-cm radial expansion and supraclavicular and mediastinal lymph node regions. Gross tumor volume was planned to receive 60 Gy (30 fractions at 2 Gy per fraction) and 40 Gy to clinical target volume (20 fractions at 2 Gy per fraction).

Icotinib was orally administrated as a 125-mg dose delivered 3 times per day concurrently with RT. Treatment was not interrupted unless stopped owing to excessive toxic effects, disease progression, or patient’s request.^22^

### Dose Modifications

Acute and late radiation toxicity was scored according to the Radiation Therapy Oncology Group morbidity scoring criteria. Irradiation was interrupted for grade 3 or higher toxicity. Radiotherapy was restarted when toxicity recovered to grade 2 or lower. A 1-week treatment break from icotinib was required for grade 3 or higher toxicity. Interruptions were allowed for up to 2 weeks. Oral icotinib was restarted at full dose when toxicity resolved to grade 2 or lower.

### EGFR Expression Assessment

Levels of EGFR expression were assessed using immunohistochemistry analysis in the central laboratory and scored independently by 2 pathologists who were blinded to clinical information using a 4-tier scoring scheme as follows: 0, no staining; 1+, faint cytoplasmic or/and membranous reactivity; 2+, moderate cytoplasmic and/or membranous reactivity; and 3+, strong cytoplasmic and/or membranous reactivity in at least 10% of tumor cells. If they could not reach agreement, a third pathologist helped with scoring. Tumors with a score of 3+ were interpreted as high expression (EGFR overexpression); others were interpreted as low EGFR expression.

### Evaluation and Follow-up

All patients were hospitalized and assessed weekly during the treatment course or more often if clinically indicated. A history and physical examination, including a complete blood cell count were performed weekly, and serum chemistry was repeated every 2 weeks for toxicity assessment. The treatment toxicity was evaluated by the Common Toxicity Criteria version 4.0 and was recorded according to the worst score achieved during treatment.

Response of the primary tumor was determined 2 months after the completion of RT. Patients were scheduled to undergo neck-thorax-abdomen computed tomography and esophagogastroduodenoscopy. Follow-up was regularly carried out at 3-month intervals in the first 2 years, at 6-month intervals for 3 years, and then annually. Assessment was performed whenever disease progression was suspected. Esophageal recurrence was confirmed by histology or cytology. Treatment failure was defined as any sign of local or regional recurrence or distant metastasis.

### Outcomes

The primary end point was OS, which was defined as the time from the date of group assignment to the date of death or the last follow-up (June 30, 2019). The secondary end points including progression-free survival (PFS) and treatment toxicity. Progression-free survival was measured from randomization until disease progression, death, or the date of the last follow-up.

### Statistical Analysis

Data were analyzed from July 1 to September 30, 2019. Sample size was estimated according to the methodology by Lakatos.^23,24^ This trial was designed to have an 80% power to detect a 20% 2-year survival improvement (from 30% to 50%) with the addition of icotinib to concurrent RT,^25^ assuming an accrual time of 1 year, a minimum 2-year follow-up, and 5% missing data. The trial needed a recruitment of 127 patients (63 in the control group and 64 in the combination treatment group) with 80% power to detect an improvement in 2-year survival (Supplement 1).

All statistical analyses were performed using SPSS, version 20.0 (SPSS Inc). Efficacy and toxicity were evaluated in the intention-to-treat population. The χ^2^ test was used to evaluate differences of patient characteristics and toxicity. Survival analysis was performed using the Kaplan-Meier method. We did a stratified log-rank comparison of OS in the intention-to-treat population. Univariate and multivariate analyses with the Cox proportional hazards model were used to investigate the prognostic factors (TKI treatment, sex, age, T stage, N stage, ECOG performance status, tumor location, and tumor length) on survival. All statistical analyses were performed with a 2-sided *P* < .05 to indicate significance.

## Results

### Patient Characteristics

**Figure 1.**
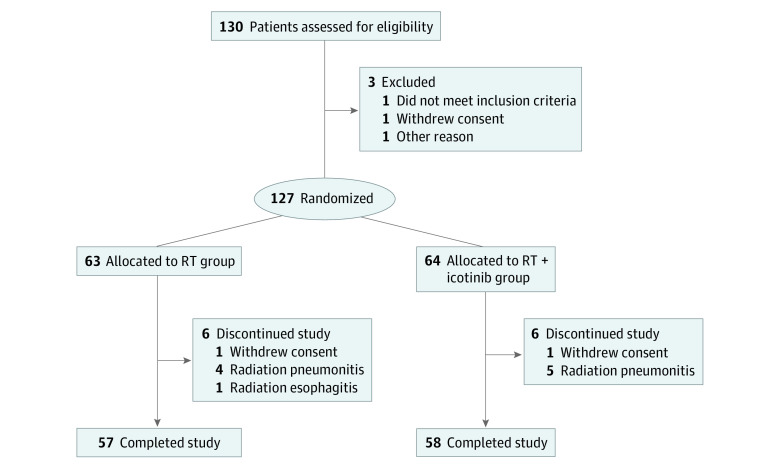
CONSORT Diagram of Participant Flow Through the Trial

Between January 1, 2015, and October 31, 2016, 127 consecutive patients with newly diagnosed ESCC were enrolled in the trial; 63 were randomized to receive RT, and 64 were randomized to RT plus icotinib (Figure 1). Detailed demographic and baseline characteristics are listed in Table 1. The median age was 76 years (range, 70-91 years), 76 patients were men (59.8%), and 51 were women (40.2%). A total of 90 patients (70.9%) were considered malnourished (weight loss >10% of body weight). The clinical stage distribution was as follows: stage II, 65 (51.2%); stage III, 55 (43.3%); and stage IVA, 7 (5.5%). No demographic difference was observed between the 2 treatment groups.

**Table 1.** Baseline Patient Characteristics

Characteristic	No. of patients (%)	
	RT (n = 63)	RT plus icotinib (n = 64)
Age, y		
≤70-74	23 (36.5)	29 (45.3)
≥75-79	23 (36.5)	22 (34.4)
≥80	17 (27.0)	13 (20.3)
Sex		
Male	36 (57.1)	40 (62.5)
Female	27 (42.9)	24 (37.5)
ECOG performance status		
0-1	36 (57.1)	31 (48.4)
2	27 (42.9)	33 (51.6)
T stage		
T2	16 (25.4)	17 (26.6)
T3	37 (58.7)	35 (54.7)
T4	10 (15.9)	12 (18.8)
N stage		
N0	25 (39.7)	27 (42.2)
N1	38 (60.3)	37 (57.8)
M stage		
M0	59 (93.7)	61 (95.3)
M1a	4 (6.3)	3(4.7)
Clinical stage (UICC 2002)		
IIA	22 (34.9)	21 (32.8)
IIB	10 (15.9)	12 (18.8)
III	27(42.9)	28 (43.8)
IVA	4 (6.3)	3 (4.7)
Tumor location		
Cervical esophagus	4 (6.3)	6 (9.4)
Upper third	17 (27.1)	14 (21.9)
Middle third	28 (44.4)	31 (48.4)
Lower third	14 (22.2)	13 (20.3)
Tumor length, cm		
<5	23 (36.5)	31 (48.4)
≥5	40 (63.5)	33 (51.6)
Weight loss in 6 mo		
≤10% body weight	22 (34.9)	15 (23.4)
>10% body weight	41 (65.1)	49 (76.6)
Dysphagia, Atkinson score^a^		
0-1	34 (54.0)	37 (57.8)
≥2	29 (46.0)	27 (42.2)

Abbreviations: ECOG, Eastern Cooperative Oncology; RT, radiation therapy.

^a^ Scores range from 0 to 4, with higher scores indicating greater dysphagia.

### Treatment Compliance

The number of patients who completed per-protocol treatment was similar (57 [90.5%] in the RT group and 58 [90.6%] in the RT plus icotinib group). Nine patients in the RT group (14.3%) vs 10 in the RT plus icotinib group (15.6%) experienced a temporary interruption of RT with a median interruption of 4 days (range, 3-6 days). Four patients in the RT group (6.3%) compared with 5 in the RT plus icotinib group (7.8%) received less than 60 Gy of radiation dose owing to grade 3 radiation pneumonitis. Termination of RT at 40 Gy for grade 4 radiation esophagitis occurred in 1 patient (1.6%) in the RT group. One patient (1.6%) discontinued icotinib after 4 weeks owing to a swallowing problem and liver function damage.

### Treatment-Related Toxicity

The incidence of treatment-related toxic effects is shown in Table 2. The majority of the adverse events were described as mild to moderate in severity. The most common toxic effects were radiation esophagitis (112 patients [88.2%]), neutropenia (69 patients [54.3%]), and radiation pneumonitis (63 patients [49.6%]) in both groups, without significant differences. Grade 3 or 4 toxic effects were observed in 17 (patients 26.6%) in the RT plus icotinib group vs 14 patients (22.2%) in the RT group (*P* = .50). No significant difference was found in radiation pneumonitis between the RT plus icotinib group (13 grade 1 cases [20.3%], 11 grade 2 [17.2%], 1 grade 3 [1.6%], and 0 grade 4) and the RT group (10 grade 1 cases [15.9%], 13 grade 2 [20.6%], and 0 grades 3 and 4) (*P* = .83); however, 1 patient in the RT group died from grade 5 radiation pneumonitis. Patients developed higher skin rash in the RT plus icotinib group (*P* = .01). Toxic effects were well controlled with appropriate dose reductions and supportive care.

**Table 2.** Treatment-Related Toxic Effects Compared Between the 2 Groups

Toxic effect	No. of patients (%)								*P* value
	RT (n = 63)^a^				RT plus icotinib (n = 64)				
	Grade 1	Grade 2	Grade 3	Grade 4	Grade 1	Grade 2	Grade 3	Grade 4	
Hematologic toxicity									
Neutropenia	14 (22.2)	18 (28.6)	2 (3.2)	0	17 (26.6)	16 (25)	2 (3.1)	0	.94
Thrombocytopenia	10 (15.9)	13 (20.6)	0	0	13 (20.3)	11 (17.2)	1 (1.6)	0	.48
Anemia	9 (14.3)	3 (4.8)	0	0	8 (12.5)	3 (4.7)	0	0	.96
Nonhematologic toxicity									
Radiation esophagitis	24 (38.1)	23 (36.5)	7 (11.1)	1 (1.6)	28 (43.8)	21 (32.8)	8 (12.5)	0	.82
Radiation pneumonitis	20 (31.7)	7 (11.1)	3 (4.8)	0	19 (29.7)	9 (14.1)	4 (6.3)	1 (1.6)	.83
Nausea/vomiting	20 (31.7)	5 (7.9)	0	0	24 (37.5)	4 (6.3)	0	0	.77
ALT/AST elevation	7 (11.1)	4 (6.3)	0	0	9 (14.1)	4 (6.3)	1 (1.6)	0	.73
Skin rash	5 (7.9)	1 (1.6)	0	0	15 (23.4)	5 (7.8)	0	0	.01
Diarrhea	6 (9.5)	2 (3.2)	0	0	9 (14.1)	4 (6.3)	0	0	.45

Abbreviations: ALT, alanine transaminase; AST, aspartate transaminase; RT, radiation therapy.

^a^ One patient (1.6%) experienced grade 5 radiation pneumonitis after a radiation of 54 Gy in the RT group.

### Survival Analysis

**Figure 2.**
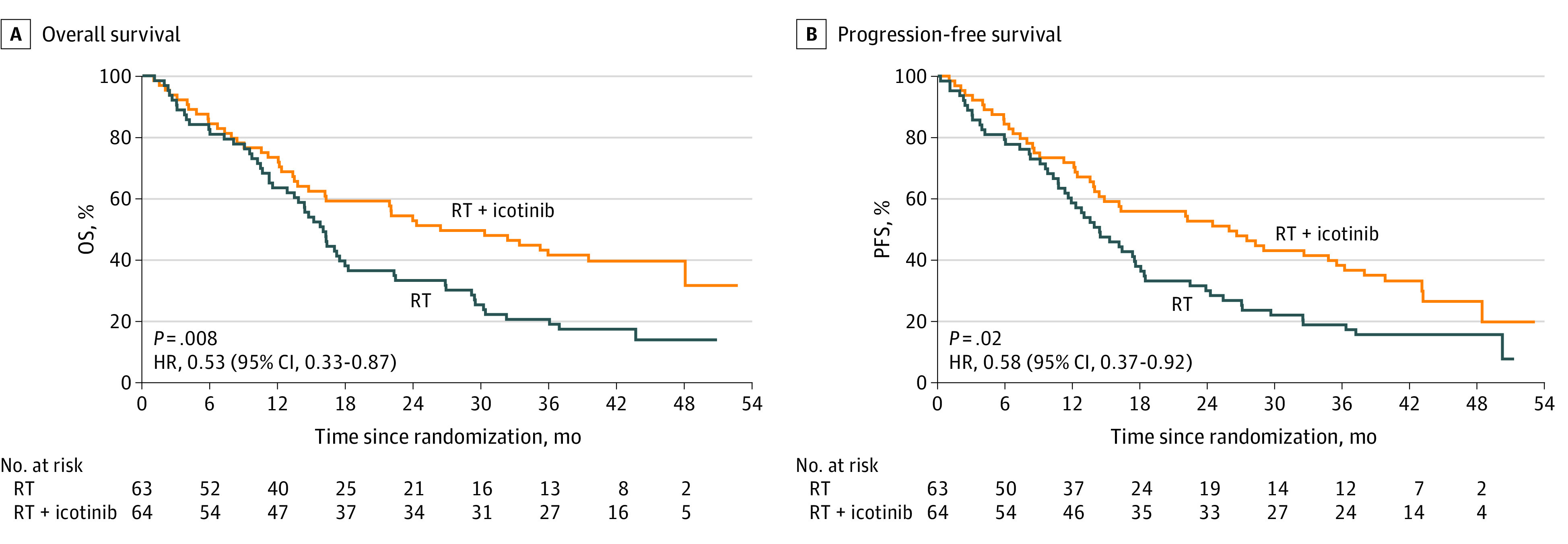
Kaplan-Meier Analyses of Survival in the Intention-to-Treat Population HR indicates hazard ratio; OS, overall survival; PFS, progression-free survival; and RT, radiotherapy.

The median follow-up time was 42.8 months (interquartile range, 42.1-43.6 months) for all patients. The overall response rate was 84.4% in the RT plus icotinib group and 60.3% in the RT group. Patients in the RT plus icotinib group had a significantly better survival compared with their RT counterparts (hazard ratio, 0.53; 95% CI, 0.33-0.87; *P* = .008. Figure 2A), with a median OS of 24.0 (95% CI, 22.2-25.8) months vs 16.3 (95% CI, 13.8-18.8) months and 2-year OS of 43.7% (95% CI, 29.2%-65.4%) vs 29.6% (95% CI, 19.4%-49.1%). Median PFS was 22.7 (95% CI, 18.3-27.1) months in the RT plus icotinib group and 14.3 (95% CI, 10.7-17.9) months in the RT group (Figure 2B). Patients in the RT plus icotinib group had a significantly longer PFS (hazard ratio, 0.58; 95% CI, 0.37-0.92; *P* = .02).

**Figure 3.**
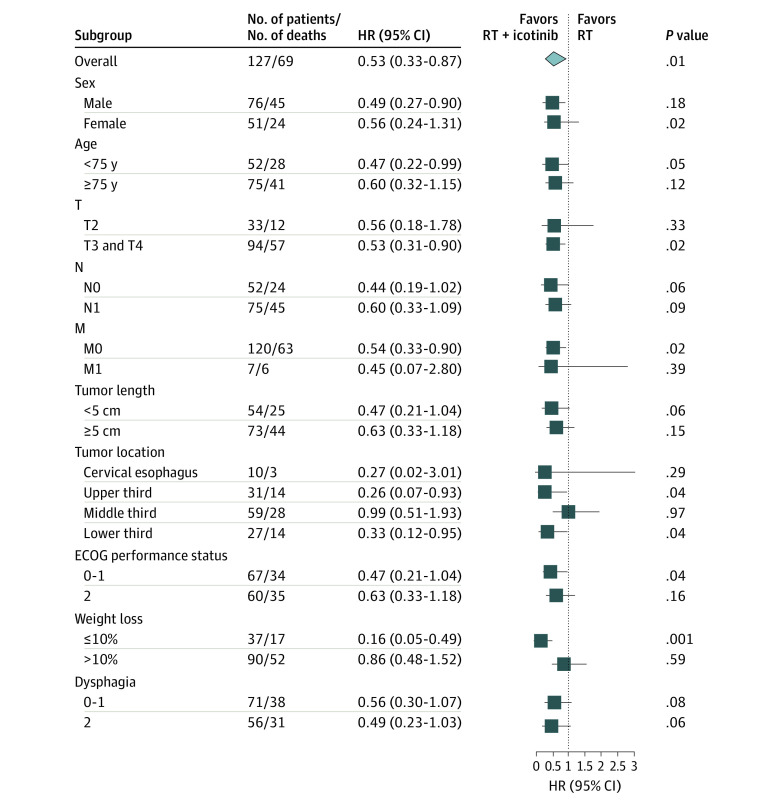
Subgroup Analysis of Icotinib Benefit for Overall Survival HR indicates hazard ratio; RT, radiotherapy.

The survival benefit of icotinib added to RT was evident in all subgroups examined (Figure 3). By univariate and multivariate analysis, use of icotinib was an independent prognostic factor (eTables 1 and 2 in Supplement 2).

### Expression of EGFR Protein

In the post hoc analysis, EGFR protein expression was evaluated in 61 patients (48.0%) from whom there were sufficient pretreatment tumor tissues for immunohistochemistry, including 32 (50%) from the RT plus icotinib group and 29 (46%) from the RT group. In the final analysis, no expression of EGFR, 1+ EGFR expression, 2+ EGFR expression, and 3+ EGFR expression were observed in 5, 6, 14, and 7 patients in the RT plus icotinib group and in 11, 2, 15, and 1 patient in RT group, respectively. In the RT plus icotinib group, median OS was not reached in the EGFR overexpression group and was 16.3 months (range, 2.6-45.1 months) in the low EGFR expression group (*P* = .03) (eFigure 1 in Supplement 2). In the RT group, median OS was 16.2 months (range, 2.6-45.1 months) in patients with low EGFR expression, and the 1 patient with EGFR overexpression lived 32.3 months with no disease progression (eFigure 2 in Supplement 2).

### Patterns of Failure

The crude patterns of primary failure are shown in eTable 3 in Supplement 2. Twenty-six patients (40.6%) in the RT plus icotinib group experienced disease recurrence compared with 41 (65.1%) in the RT group. The incidence of local or regional recurrence was significantly lower in the RT plus icotinib group (26 patients [40.6%]) than the RT group (38 patients [60.3%]) (*P* = .03). Adding icotinib to RT diminished the rate of distant metastasis (6.3% vs 15.9%, respectively; *P* = .08), but this finding was not statistically significant.

## Discussion

Because of their reduced physiologic reserve of body function and highly prevalent comorbidities, older patients have poor tolerance for CCRT and are at increased risk of toxic effects. Therefore, palliative RT has been generally recommended. Modern approaches to cancer treatment focus on combination strategies involving targeted therapy and RT. The essential hypothesis of the present trial was that icotinib concurrently administered with RT would promote local and regional effects as well as diminish distant metastases.

Our trial found that patients who received concurrent icotinib with RT had significantly longer OS and PFS than those who received RT alone. The OS benefit persisted across all subgroups. The available data suggest that the combination resulted in not only less local and regional recurrence but also fewer distant metastases at 2 years.

Few studies have reported the application of RT plus EGFR TKIs in older patients with esophageal cancer.^26,27^ The combination of gefitinib plus RT for elderly patients was reported by Xu and colleagues.^26^ Their results showed a median OS of 14.0 months, with 5 of 20 patients experiencing severe toxic effects.^26^ In a pilot study^18^ evaluating the efficacy of concurrent erlotinib and RT for chemoradiotherapy-intolerant patients with ESCC, the median OS was 21.1 months, which is similar to our experimental group. Inconsistent with our results, RTOG 0436 trial^13^ failed to demonstrate the superiority of the addition EGFR inhibitor to CCRT. We consider that this negative result may be partly due to the higher incidence of toxicity caused by the intensified treatment with CCRT and cetuximab. In addition, unlike our study, RTOG 0436 enrolled patients with a mixture of squamous cell cancer and adenocarcinoma, which may weaken the benefits of cetuximab.^13^

Median survival and 2-year survival in the RT group in our trial was higher compared with the RT-alone arm of RTOG 85-01 (16.3 vs 8.9 months, 29.6% vs 10%).^28^ This finding may be partly owing to the progress of modern technique and greater experience in radiation management. Our results that favored adding icotinib to RT did not depend on worsened data in the control group.

The ARTDECO study^29^ explored definitive radiotherapy concurrent with carboplatin and paclitaxel in locally advanced esophageal cancer and demonstrated a 3-year local progression-free interval of 77% for ESCC and a 3-year OS rate of 40%. The median age of these patients was 70 years. These findings suggested the safety and tolerability of CCRT in older patients. However, in our study, enrolled patients were older than in the ArtDeco trial. In the present study, more than 70% of patients were malnourished. These differences may contribute to the difference in CCRT tolerance.

Our study showed that this combined therapy was generally well tolerated in patients with ESCC. The toxic effects associated with icotinib and RT did not overlap. The addition of icotinib did not increase other toxic reactions except for mild skin rash and diarrhea. The safety profile of icotinib observed in this trial was consistent with that seen previously with EGFR TKIs for the treatment of esophageal cancer.^17,30^ The most common radiation toxic effects were esophagitis and pneumonitis, which were similar to historical data.^30^ Few cases of grade 3 or 4 hematologic toxic effects were observed in our trial. However, grade 3 or 4 hematologic toxic effects have been commonly reported in patients treated with CCRT.^4,6^ No significant difference was found in radiation pneumonitis between the RT plus icotinib group and RT group. This finding is in accordance with previous reports.^31,32,33^ Therefore, the combination of icotinib and RT had a more favorable toxicity profile than did CRT.

A previous study has evaluated the feasibility of icotinib in patients with advanced ESCC with EGFR overexpression.^17^ The response rate was higher (17.6% vs 0%, *P* = .34) for patients with high EGFR-expressing tumors. Furthermore, all patients who responded to icotinib showed EGFR overexpression. Another study suggested that EGFR amplification appears to identify a subgroup of patients with esophageal cancer who may benefit from gefitinib.^34^ Few studies evaluated the association between EGFR expression and survival parameters in EGFR TKIs combined with RT.^35^ Our study found that in the RT plus icotinib group, patients with EGFR overexpression had a significantly better OS (not reached vs 16.3 months, *P* = .03). In addition, EGFR expression could help identify patients who will benefit more from the combination of EGFR TKIs and RT.

### Limitations

Our study has some limitations. Tumor tissues were obtained from only 61 patients for EGFR expression assessment. Analysis of EGFR expression was conducted with a limited sample size, and we did not include EGFR status as part of the eligibility criteria. We analyzed the data at a median follow-up of approximately 2 years. Long-term follow-ups are warranted to evaluate the toxic effects and efficacy. Icotinib is not currently available outside China.

## Conclusions

The findings of this study suggest that concurrent therapy with icotinib and RT was well tolerated and tended to improve survival in older patients with ESCC. Patients with EGFR overexpression seemed to benefit more from icotinib with RT. A phase III trial to further identify the efficacy of icotinib with RT is forthcoming.

## Notes

Supplement 1. **Trial Protocol**

Supplement 2. **eFigure 1.** Overall Survival in RT + Icotinib Group **eFigure 2.** Overall Survival in RT Group **eTable 1.** Univariate Analysis of Potential Factors Associated With Survival in all ESCC Patients **eTable 2.** Multivariate Analysis of Prognostic Factors in all ESCC Patients **eTable 3.** Patterns of First Failure Between Two Groups of Elderly ESCC Patients

Supplement 3. **Data Sharing Statement**
