# Supplementary material for: Icotinib With Concurrent Radiotherapy vs Radiotherapy Alone in Older Adults With Unresectable Esophageal Squamous Cell Carcinoma: A Phase II Randomized Clinical Trial
Source: JAMA Netw Open. 2020 Oct 7;3(10):e2019440. doi: 10.1001/jamanetworkopen.2020.19440 (PMC7542309; doi:10.1001/jamanetworkopen.2020.19440)
Supplement: Supplement 1. — Trial Protocol [file jamanetwopen-e2019440-s001.pdf]

CLINICAL PROTOCOL

Icotinib with Concurrent Radiotherapy versus Radiotherapy alone in Elderly Patients with Esophageal Squamous Cell Carcinoma: A Randomized Multicenter Open-label Phase II Trial

Test product: Icotinib
Study Sponsor: Hangzhou Cancer Hospital Major Subject Foundation and Zhejiang Bata Pharma Inc. China
Principal Investigator: Prof Shixiu Wu, M.D.
Hangzhou Cancer Hospital
34 Yanguan Lane, Hangzhou
Recruitment Site: Hangzhou Cancer Hospital, Hangzhou, China; Huaian First People's Hospital, Huaian, China; Lishui Central Hospital, Lishui, China.

Activation Date: **December 12，2014**
Update Date: **December 22, 2014**
Version Date: **January 12, 2015**

TABLE OF CONTENTS

1 BACKGROUND AND RATIONALE
  1.1 Unmet Medical Needs
  1.2 A Novel Therapeutic Stratagy for Elderly Patients with ESCC
  1.3 Study Rationable
2 STUDY OBJECTIVES
  2.1 Primary Endpoints of the Study
  2.2 Secondary Endpoints of the Study
  2.3 Exploratory Endpoints of the study
3 STUDY DESIGN
  3.1 Study Population
  3.2 Screening of Participants
4 STUDY PROCEDURES
  4.1 Subject Enrolment
  4.2 Treatment Procedures
5 DESCRIPTION OF STUDY
  5.1 Common Toxicity Criteria
  5.2 Concomitant Treatments
  5.3 Treatment Interruptions
  5.4 Dose Modifications
  5.5 EGFR Expression Assessment
  5.6 Schedule Visits and Follow-Up
  5.7 Withdrawal of Study Subjects
  5.8 Lost to Follow-Up
  5.9 Assessments
6 SAFETY DATA COLLECTION, RECORDING, AND REPORTING
  6.1 Adverse Events
  6.2 Serious Adverse Events
7 END OF STUDY
  7.1 Primary Completion
  7.2 End of Trial
8 STATISTICAL CONSIDERATIONS
  8.1 Study Endpoints, Subsets, and Covariates (Phase II)
9 ETHICAL CONSIDERATIONS
  9.1 Good Clinical Practice
  9.2 Independent Ethics Committee
  9.3 Informed Consent
  9.4 Subject Confidentiality
10 REFERENCES

1. BACKGROUND AND RATIONALE

1.1 Unmet Medical Needs

Esophageal cancer is the sixth most common cause of cancer deaths worldwide[1] .Esophageal cancer increases in incidence with age. It is estimated that over 20% of patients with esophageal cancer are diagnosed at an elderly age[2]. The number of elderly patients with esophageal cancer is expected to increase in the future.

Surgery plays the pivotal role for the treatment of early-stage and localized esophageal carcinoma[3]. For patients with localized esophageal carcinoma selected for nonsurgical treatment, the standard therapy is concurrent chemoradiation therapy[4,5]. However, due to the limitations of medical conditions, the elderly patients with esophageal carcinoma are generally not considered candidates for surgery and are unable to tolerate the toxicity of concurrent chemoradiation[3,6,7].Palliative radiotherapy alone should generally be recommended for elderly patients, but with a limit survival benefit[4]. There is an urgent need to develop novel agents with a favorable toxicity profiles for elderly patients with localized esophageal carcinoma.

1.2 A Novel Therapeutic Stratagy for Elderly Patients with esophageal carcinoma

Epidermal growth factor receptor (EGFR) is overexpressed in 30–70% of esophageal carcinoma cases and is associated with poor prognosis and an inferior response to conventional treatment[8,9]. EFGR tyrosine kinase inhibitors (TKIs) achieved response rates of 2% to 13% in advanced squamous cell carcinoma and adenocarcinoma of the esophagus in phase II trials[10,11]. EGFR TKI helps disrupt cell growth pathways and makes cells more sensitive to the effects of radiotherapy[12,13]. EGFR TKI combined with chemoradiotherapy (CRT) has been evaluated in esophageal carcinoma[14] . EGFR TKIs plus thoracic radiation have confirmed the safety profile and have shown some clinically beneficial in esophageal cancer patients[15-17]. Elderly patients with esophageal cancer may benefit from EGFR TKIs plus thoracic radiation. In an attempt to improve the clinical results, a pilot study of erlotinib plus radiotherapy in elderly esophageal cancer patients showed encouraging survival results and reasonable safety profile[16].

1.3 Study Rationable

A recent Chinese study showed that for elderly patients received oral gefitinib with RT, the overall response rate was 90%. Median OS and progression free survival were 14.0 and 7.0 months, respectively[18]. Iyer et al. evaluated the combination of erlotinib plus RT for patients aged over 65 years in USA[15]. Survival results revealed that the median OS and progression free survival were 7.3 and 4.5 months, respectively.

Icotinib, another oral EGFR-TKI, was associated with more favorable toxicity profile than gefitinib or erlotinib[8]. It was reported that icotinib markedly inhibited the proliferation of the human epidermoid squamous carcinoma A431 cell line with a high level of EGFR[8]. The toxicities associated with icotinib and RT do not overlap, which enables their concomitant administration. There were no direct comparisons between radiotherapy alone and radiotherapy plus icotinib for esophageal cancer. However, a prospective phase I study showed that the combination of icotinib and IMRT in patients with locally nasopharyngeal cancer had an acceptable safety profile and was well tolerated[19].

2. STUDY OBJECTIVES

2.1. Primary Endpoints of the Study

- To evaluate whether overall survival in the combined group was superior to the RT alone group.

2.2. Secondary Endpoints of the Study

- To evaluate disease free survival and treatment toxicity.

2.3. Exploratory Objective

• To evaluate the predictive biomarkers in subjects receiving icotinib treatment.

3. STUDY DESIGN

This is a randomized, multicenter, open-label phase II trial to evaluate the efficacy and toxicity of radiotherapy plus epidermal growth factor receptor (EGFR) tyrosine-kinase inhibitor (icotinib) in elderly esophageal squamous cell carcinoma (ESCC) patients. The study will consist of 3 periods: screening period (up to 4 weeks); treatment period (6 weeks); follow-up period (3-month interval in the first 2 years, at 6-month interval for 3 years, then annually). The trial protocol was approved by the institutional review board and independent ethics committee of each participating center (ClinicalTrials.gov, study identifier: NCT02375581). Patients will complete the scheduled treatment until disease progression or until discontinuation of study therapy (discontinuation due to toxicity, withdrawal of consent).

3.1. Study Population

Signed informed consent was obtained before any study procedure. Patients must be able to understand and be willing to sign a written informed consent.

Inclusion/exclusion criteria must be met at the time of study screening.

3.1.1 The Criteria for inclusion in our study included:

1) Pathologically confirmed esophageal squamous cell cancer;

2) Clinical stages II–IVA disease according to the International Union Against Cancer (UICC, 2002) TNM stage criteria, without contraindication for radical radiotherapy;

3) Aged ⩾ 70;

4) Eastern Cooperative Oncology Group (ECOG) performance status 0-2；

5) adequate bone marrow, renal, hepatic, cardiac, and respiratory function（In 7 days after being screened, subjects should follow the status: WBC ⩾ 3.0 x 10^9/L; ANC ⩾ 1.5x 10^9/L; PLT ⩾ 80 x 10^9/L; Hb ⩾ 90 g/L; serum Cr ⩽ ULN; serum bilirubin ⩽ 1.5 ULN; ALT/AST ⩽ 1.5 ULN）;

6）Subjects should sign for the informed consent;

7）Subjects should perform good compliance;

8）No obstacle in oral medicine .

3.1.2 The exclusion criteria were as follows:

1) Patients who have or are currently undergoing additional chemotherapy, radiation therapy or targeted therapy;

2) Complete obstruction of the esophagus, or patients who have the potential to develop perforation;

3)Patients with a history of malignancy (except that skin carcinomas or in situ breast cancer, oral cancer and cervical cancer with expected survival ⩾2 years);

4) Patients who have multiple foci esophageal carcinomas;

5) Patients who are/were given any other medicine tests currently/in last 4 weeks;

6) Experienced hypersensitiveness with similar medicine or other kinds of bio-medicines;

7) Patients who have complications as following:

a) Uncontrolled angina and heart failure, have a history of hospitalization in 3 months;

b) A history of myocardial infarction in the past 6 months;

c) There is a need for antibiotic treatment of acute bacterial or fungal infection;

d) Chronic obstructive pulmonary disease, or other lung disease requiring hospitalization;

e) Drug addiction, alcoholism and AIDS disease or long-term virus carriers;

f) Uncontrollable seizures, or loss of insight because of mental illness.

3.2 Screening of Participants

Medical history will be collected during screening and must date back to the original diagnosis of ESCC. If a subject is referred to the study center, a copy of all applicable reports and histological evidence confirming the diagnosis must be provided to the study center before enrolment. Once a patient has personally signed the consent form, study specific screening procedures may commence.

All patients must have the following procedures within 2 weeks before their therapy (imaging examination within 4 weeks):

- Review and documentation of inclusion and exclusion criteria.
- Medical and medication history
- Height and weight
- ECOG performance status
- Physical examination
- 12-lead electrocardiogram (ECG) taken after the patient has been supine for at least 5 minutes
- Laboratory tests: blood chemistry, full blood count, coagulation parameters, stool test, urinalysis, calculated creatinine clearance
- Imaging by neck/chest CT, abdominal/ pelvic CT/MRI to assess disease extent
- FDG-PET/CT scan, brain CT/MRI and cardiac ultrasound as clinical indicated
- Immunohistochemistry to analyze EGFR expression in patients were optional, but encouraged.

4. STUDY PROCEDURES

4.1. Subject Enrolment

Before patients are enrolled into the study, a copy of an approval letter clearly identifying the version of the protocol and patient informed consent form are required. All patients must personally sign and date the consent form before any study-specific screening procedures are performed. Standard medical practice procedures performed prior to the signing of the consent form, which are performed irrespective of the patient's potential involvement in the study, may be used for screening purposes if they comply with the study requirements. The expectation is that once the informed consent form is signed, study specific screening procedures will commence as soon as is practicable.

Patients who do not meet the eligibility criteria may be rescreened at the discretion of the investigator if their status changes. However, once enrolled, the patient may not be enrolled more than once in the study.

4.2. Treatment Schedule

Eligible patients were randomly assigned, in a 1:1 ratio, to two groups: RT+icotinib group and RT alone group. Central randomization was done with the use of the random number table method. Patients were stratified according to clinical stage.

4.2.1 Radiotherapy

Radiotherapy consisted of 60 Gy given in 30 fractions in all patients using high-energy linear accelerators with conventional fraction, three-dimensional conformal technique or intensity-modulated radiotherapy (3D-CRT/IMRT).

For both newly diagnosed patients in two groups, the gross tumor volume (GTV) was defined as the volume of the primary tumor shown on the esophageal barium exam, upper digestive endoscopy and CT; and metastatic lymph nodes were defined as lymph nodes of ≥1 cm in the shortest axis and ≥5 mm local in the trachea oesophagus groove. The clinical target volume (CTV) was defined by adding 5-cm margins of proximal and distal uninvolved oesophagus, including 1-cm lateral margins. The

planning target volume (PTV) was calculated by adding 1-cm margins around the CTV, but the margins were reduced when the PTV recovered the spinal cord. The gross tumor volume (GTV) received 60 Gy (30 fractions at 2 Gy per fraction) and clinical target volume (CTV) was 40 Gy (20 fractions at 2 Gy per fraction).

Plan optimisation was as follows: (1) 99% of the PTV was covered by 95% of the prescribed dose; (2) 95% of the PTV volume was covered by the prescribed dose; and (3) the maximum dose did not exceed 110% of the prescribed dose in a continuous volume of <1 cm3 in the PTV.

The normal tissue constraints of the critical organs were as follows: the maximum spiral cord point dose of ≤45 Gy; the lung V20 (percentage of total lung volume receiving ≥20 Gy) ≤ 30%, and the mean lung dose ≤ 16 Gy; the mean heart dose ≤ 30 Gy; and the maximum intestine dose ≤ 50 Gy.

4.2.2 Icotinib Treatment

Icotinib was orally administrated at a dose of 125mg/time, three times a day, during the course of radiotherapy in RT+icotinib group without interruption until it was stopped due to excessive toxicity, disease progression, or patient request.

5. DESCRIPTION OF STUDY

This phase II study foresees a total of 6 weeks of treatment. In the study, all patients will receive radiotherapy at a dose of 60Gy. In the RT +icotinib group, icotinib will be administered concurrently with RT.

5.1. Common Toxicity Criteria

Acute and late radiation toxicity was scored according to the Radiation Therapy Oncology Group (RTOG) morbidity scoring criteria. The icotinib toxicity was evaluated according to the common toxicity criteria for National Cancer Institute Common Terminology Criteria for Adverse Events version 4.0 (NCI-CTAE 4.0). Toxicities were recorded according to the worst score achieved during treatment.

5.2. Concomitant Treatments

All medication administered must be recorded in the CRF (e.g., Concomitant Treatment Summary CRF) including the name, dose, possible side effects, start and if applicable stop date. Concomitant medication administered during the study must be recorded in the CRF including the generic name, dose, possible side effects, and start and end date of therapy. Any change in documented, concomitant medication must also be recorded.

5.3. Treatment Interruptions

Therapy interruptions will usually not be necessary. However, if radiation is held for any reason, all Concurrent therapy must also be held. Interruptions may be kept to a minimum by the use of ancillary therapy and vigorous nutritional support.

Interruptions are permitted only on the basis of toxicity.

- Non-hematologic toxicities

1) Grade 3 or 4 radiation esophagitis or radiation pneumonitis.
2) Clinically significant grade 3 or 4 biochemical abnormalities.

- Haematological toxicities

1) Febrile neutropenia (absolute neutrophil count (ANC) < 1 × 109/L and fever > 38.5℃).
2) Grade 3/4 neutropenia.
3) Grade 3/4 thrombocytopenia.

- Hepatic toxicities

1) ALT or AST > 5 ULN.
2) Total bilirubin > 3 ULN.

- Grade 3/4 skin rash or diarrhea
- Episodes of vomiting (≥ grade 3) lasting ≥ 3 days and unresponsive to antiemetics
- Weight loss ≥ 10% (≥ grade 2) of pretreatment weight.

Rarely, nontreatment related or unexpected toxicities may require interruption of therapy at the discretion of the treating oncologist. Interruption of therapy may continue until the toxicity has regressed to ≤grade 2 to allow resumption of therapy; however, every effort should be made to limit treatment interruptions to 1-2 weeks.

5.4. Dose Modifications

Dose modifications were considered on a weekly basis during the treatment. Acute and late radiation toxicity was scored according to the Radiation Therapy Oncology Group morbidity scoring criteria. Every effort must be made to deliver the full dose to all patients. Irradiation was interrupted for grade 3 or higher toxicities. Once the toxicities recovered to ≤ grade 2, the patient's therapy should resume and full protocol dose should be delivered. The toxicity that forced any dose reduction must be documented. If interruption of therapy (up to 2 weeks) becomes necessary, radiation therapy should be completed to the prescribed doses. Total number of fractions and elapsed days should be carefully reported. If an interruption of more than 2 weeks is necessary, resumption of treatment is at the discretion of the radiation oncology chairs. The patient's treatment plan will be considered a major deviation, but follow-up will be continued. If a patient develops grade 3 esophagitis in the last week of treatment (i.e., with 5 or fewer radiation treatments remaining), radiation therapy may continue at the discretion of the treating physician.

The National Cancer Institute's Common Toxicity Criteria version 4.0. were used for toxicity of icotinib. A 1-week treatment break from icotinib was required when grade 3 or higher toxicity was observed. Interruptions were allowed up to 2 weeks. Oral icotinib was restarted at full dose when toxicities recovered to ≤ grade 2.

5.5. EGFR Expression Assessment

Levels of EGFR expression were assessed using immunohistochemistry (IHC) analysis in the central laboratory. All tissue samples were immersed and fixed in 4% paraformaldehyde, embedded in paraffin, and sectioned at a thickness of 8 μm. Sections were probed with anti-EGFR antibodies and then with secondary antibodies. The sections were counterstained with hematoxylin and viewed under a microscope to assess the percentage and intensity of nuclear and non-nuclear staining in tumor cells as well as background staining.

5.6. Schedule Visits and Follow-Up

5.6.1 Schedule Visits

1) All patients must have the following procedures completed at selected times.

Recording of adverse events and changes to adverse events.

Recording of changes to concomitant medication.

ECOG performance status.

Physical examination.

12-lead ECG.

Laboratory tests: blood chemistry, full blood count, coagulation parameters, calculated creatinine clearance.

Barium oesophagram, Neck/chest CT, abdominal/ pelvic CT/MRI to assess response.

Endoscopy and ultrasonography as clinically indicated.

2) Every effort should be made to schedule visits within the timeframe stated in the protocol. In the case that the visits cannot be within the timeframe stated in protocol then the treatment period study procedures can be performed ± 2 days of the scheduled visit.

5.6.2 Follow-up

1) All subjects that discontinue treatment and have not withdrawn full consent to participate in the study will continue in the long-term follow-up phase. Long-term follow-up was regularly carried out at 3-month interval in the first 2 years and at 6-month interval for 3 years, then annually.

2) Contact will be made approximately from the subject's last administration of study treatment (for the purposes of survival assessment) and can be made by telephone call, clinic visit, through another physician or via registry search.

5.7. Withdrawal of Study Subjects

Subjects have the right to withdraw fully or partially from the study at any time and for any reason. His or her future medical care should be taken without prejudice by the physician or at the institution.

Subjects who request to discontinue the study must continue to be followed for protocol specified follow-up procedures. The only exception to this is when a subject specifically withdraws consent for any further contact with him/her.

Withdrawal of full consent for a study means that the subject does not wish to receive further investigational treatment and does not wish to or is unable to continue further study participation. Withdrawal of partial consent for a study means that the subject does not wish to take investigational product(s) any longer but is still willing to collaborate in providing further data by continuing on study (e.g., participate in all subsequent study visits or procedures and/or provide long-term follow-up/survival information).

The withdrawal of consent should be explained in detail in the medical records by the investigator and entered on the appropriate CRF page.

5.8. Lost to Follow-Up

All reasonable efforts must be made to locate subjects to determine and report their ongoing status. Lost to follow-up is defined by the inability to reach the subject after a minimum of three documented phone calls, faxes, or emails as well as lack of response by subject to one registered mail letter.

All attempts should be documented in the subject's medical records. If after all attempts, the subject remains lost to follow-up, then the last known alive date as determined by the investigator should be reported and documented in the subject's medical records. If it is determined that the subject has died, the site will use permissible local methods to obtain the date and cause of death.

5.9. Assessments

5.9.1 Safety Assessment

All patients were hospitalized and assessed weekly during the treatment course or more often if clinically indicated.

Safety assessments will be based on the following: clinical laboratory tests, ECOG performance status, physical examination including vital signs and ECG, and adverse events (AEs).

Laboratory tests will be performed by the local laboratory. Results of clinical laboratory tests performed on Day 1 of every week must be available prior to radiation dosing. Blood chemistry will include sodium, potassium, calcium, magnesium, phosphate, glucose, cholesterol, triglycerides, albumin, total protein, total bilirubin, alkaline phosphatase, ALT, AST, blood urea nitrogen, creatinine, uric acid and C-reactive protein.

Blood counts will include haemoglobin, haematocrit, white blood cells, neutrophils, basophils, eosinophils, lymphocytes, monocytes and platelets.

Results of all laboratory tests required by this protocol must be provided to investigator, recorded on the laboratory pages of the CRF.

5.9.2 Efficacy Assessment

Response of the primary tumor was determined 2 months after the completion of radiotherapy. Patients were scheduled to have endoscopy and CT/MRI scan. Once patients developed disease progression during treatment, the assessment was performed at the time of progression.

Computed tomography/magnetic resonance imaging (CT/MRI) for neck, chest, abdomen, and pelvis will be performed at screening and as clinically indicated.

CT scans should be acquired with ≤5mm slices with no intervening gap (contiguous). Should a subject have a contraindication for CT IV contrast, a non-contrast CT of the neck and chest and a contrast enhanced MRI of the abdomen and pelvis may be obtained. MRI should be acquired with slice thickness of ≤5 mm slices. Every attempt should be made to image each subject using an identical acquisition protocol on the same scanner for all imaging time points.

Brain CT or MRI scans during on-study treatment and follow up periods are required only if there is a prior history of lesions present at Screening, or as clinically indicated for new signs and symptoms that suggest CNS involvement.

PET/CT scan if there is a reasonable assumption that the patient may have occult lesions or contraindication of CT or MRI contrast.

5.9.3 On Study Procedural Outline

Table 1: Screening Procedural Outline

Procedure	Screening Visits	Notes
Day	≤ -14	
Informed Consent	×	A subject is considered enrolled only when a protocol specific informed consent is signed.
Inclusion/Exclusion Criteria	×	
Medical History	×	Baseline signs and symptoms are those that are assessed within 2 weeks prior to the treatment. Include any toxicities or allergy related to previous treatments.
Medication History	×	
Weight and Height	×	
Physical Examination	×	
ECOG performance status	×	
ECG	×	

Laboratory Tests	×	blood chemistry, full blood count, coagulation parameters, stool test, urinalysis, calculated creatinine clearance.
CT/MRI (neck, chest, abdomen, pelvis)	×	Within 28 days of study procedure on Day 1. Baseline imaging done as part of the subject's previous routine care before signing the informed consent form and completed within 28 days prior to study procedure need not be repeated.
Monitor for Adverse Events	×	All adverse events must be collected from the date of subject's written consent until 30 days post discontinuation of dosing.
FDG-PET/CT Scan		as clinical indicated
Brain CT/MRI		as clinical indicated
Cardiac Ultrasound		as clinical indicated
Tumor Tissue		Optional, but encouraged. To test EGFR expression

Table 2: On Therapy and Follow-up Procedures

parameter	Day 0	Weekly during therapy	2 months after completion of therapy	Survival follow-up
Medical History	×	×	×	×
Physical Examination	×	×	×	×
Weight*	×		×	
Full blood count	×	×	×	×
Blood chemistry*	×		×	×
Endoscopy#				
Neck, chest and abdominal CT/MRI			×	×
Ultrasonography#				
Barium oesophagram	×		×	×
ECG	×		×	×
ECOG Performance status	×	×	×	×
Adverse Events	baseline	Continuous		

*repeated every 2 weeks during treatment, # as clinically indicated.

6. SAFETY DATA COLLECTION, RECORDING, AND REPORTING

6.1. Adverse Events

6.1.1 Definition of Adverse Events

An adverse event is defined as any untoward medical occurrence in a clinical trial subject. The event does not necessarily have a causal relationship with study treatment. The investigator is responsible for ensuring that any adverse events observed by the investigator or reported by the subject are recorded in the subject's medical record and reported as described in Section 6.1.2 and Section 6.2.2.

The definition of adverse events includes worsening of a pre-existing medical condition. Worsening indicates the pre-existing medical condition (e.g., diabetes, migraine headaches, gout) has increased in severity, frequency, and/or duration, and/or has an association with a significantly worse outcome. A pre-existing condition that has not worsened during the study, and involves an intervention such as elective cosmetic surgery or a medical procedure while on study, is not considered an adverse event. Disease progression of the recurrent tumor should not be captured as an adverse event (including fatal adverse event). If there are signs and/or symptoms of disease progression that are new or worsened from baseline signs and/or symptoms, these should be reported as adverse event(s). If a new primary malignancy appears, it will be considered an adverse event.

6.1.2 Reporting Procedures for Adverse Events

The investigator is responsible for ensuring that all adverse events observed by the investigator or reported by the subject that occur after signing of the informed consent through the safety follow-up are reported using the applicable CRF (e.g., Adverse Event Summary CRF). Adverse events will not be collected for screen failures or subjects in long term follow-up for survival.

The investigator must assign the following adverse event attributes:

- Adverse event diagnosis or syndrome(s), if known (if not known, signs or symptoms).
- Dates of onset and resolution.
- Severity (and/or toxicity per protocol).
- Assessment of relatedness to icotinib or radiotheray.
- Action taken.

The adverse event toxicity grading scale used will be the CTCAE 4.0 and RTOG criteria

The investigator must assess whether the adverse event is possibly related to icotinib or radiotheray. This relationship is indicated by a "yes" or "no" response to the question: "Is there a reasonable possibility that the event may have been caused by icotinib or radiotheray?"

The investigator must assess whether the serious adverse event is possibly related to any study-mandated screening procedure. This relationship is indicated by a "yes" or "no" response to the question: "Is there a reasonable possibility that the event may be related to screening procedures?"

The investigator must assess whether the adverse event is possibly related to any study mandated activity or procedure. This relationship is indicated by a "yes" or "no" response to the question: "Is there a reasonable possibility that the event may have been caused by a study activity/procedure?"

The investigator is responsible for reviewing laboratory test results and determining whether an abnormal value in an individual study subject represents a clinically significant change from the subject's baseline values. In general, abnormal laboratory findings without clinical significance (based on the investigator's judgment) should not be recorded as adverse events. However, laboratory value changes that require treatment or adjustment in current therapy are considered adverse events. Where applicable clinical sequelae (not the laboratory abnormality) should be recorded as the adverse event.

Medically significant adverse events considered related to the investigational product by the investigator or the sponsor will be followed until resolved or considered stable. The investigator’s clinical judgment will be used to determine whether a subject should be removed from treatment or from the study due to an adverse event. A subject, or subject’s legal guardian, may also voluntarily withdraw from treatment or from the study due to an adverse event. If the subject withdraws full consent, the subject should be encouraged to undergo, at a minimum, an end-of-study assessment.

6.2. Serious Adverse Events

6.2.1 Definition of Serious Adverse Events

A serious adverse event is defined as an adverse event that meets at least 1 of the following serious criteria:

- fatal;
- life-threatening (places the subject at immediate risk of death);
- requires in-patient hospitalization or prolongation of existing hospitalization;
- results in persistent or significant disability/incapacity;
- congenital anomaly/birth defect; and/or
- other medically important serious event.

An adverse event would meet the criterion of “requires hospitalization”, if the event necessitated an in-patient admission to a health care facility (e.g., overnight stay).

Overnight stays in the hospital induced by geographical factors won’t be registered as hospitalization.

If an investigator considers an event to be clinically important, but it does not meet any of the other serious criteria, the event could be classified as a serious adverse event under the criterion of “other medically important serious event”. Examples of such events could potentially include, but are not limited to, allergic bronchospasm, convulsions, pneumonitis, treatment-induced liver injury, exfoliative dermatitis, or events that necessitate an emergency room visit, outpatient surgery, or urgent intervention.

6.2.2 Reporting Procedures for Serious Adverse Events

The investigator is responsible for ensuring that all serious adverse events observed by the investigator or reported by the subject (including screen failures) that occur after signing of the informed consent through the safety follow-up visit are recorded in the subject’s medical record.

Serious adverse events will not be collected for subjects in long term follow-up for survival.

New information relating to a previously reported serious adverse event must be reported to the principal investigator within 1 working day of receipt. The investigator may be asked to provide additional follow-up information, which may include a discharge summary or extracts from the medical record. Information provided about the serious adverse event must be consistent with that recorded on the applicable CRF (e.g., Adverse Event Summary CRF). Relevant medical records are to be submitted to principal investigator as soon as they become available; autopsy reports can and should be provided for deaths, if available.

Serious adverse events deemed possibly related to the investigational drug by the investigator occurring up to 30 days after the safety follow-up will be reported to Zhejiang Bata Pharma Inc. and the principal investigator within 1 working day of discovery or notification of the event.

If a subject is permanently withdrawn from the study because of a serious adverse event, this information must also be immediately reported.

7. END OF STUDY

7.1. Primary Completion

The primary completion of each part of the study will occur when target enrolment is complete and each subject does at least one of the following.

- Has had the opportunity to complete the safety follow-up visit. This visit must be performed 90 days after the last therapy. The safety follow-up visit completion date is the date that the final assessment and/or procedure is performed.
- Withdraws from study.

7.2. End of Trial

The end of trial will occur when target enrolment is complete and each subject either withdraws from study or completes long-term follow-up.

All subjects that have not withdrawn full consent to participate in the study will continue in the long-term follow-up phase. Long-term follow-up will continue approximately every 3 months in the first 2 years, every 6 months for 3 years, and then annually.

8. STATISTICAL CONSIDERATIONS

This is a randomized, multicenter, open-label phase II trial to evaluate the efficacy and toxicity of radiotherapy plus icotinib in elderly ESCC patients.

8.1. Study Endpoints, Subsets, and Covariates (Phase II)

8.1.1 Primary and Secondary Endpoints

Primary endpoints of the study:

To evaluate whether overall survival in the combined group was superior to the RT alone group.

Secondary endpoints of the study:

To evaluate the toxicity and disease free survival, and EGFR expression in the radiotherapy plus icotinib group.

Overall survival was calculated from the day of randomization until death or until the last follow-up.

Disease free survival was measured from the day of randomization until disease progression, death, or the date of the last follow-up for those still alive.

8.1.2 Analysis Subsets

Safety Analysis Set

The analysis of all endpoints, unless noted otherwise, will be conducted on the intention-to-treat (ITT) set. ITT is defined as all subjects that are enrolled and receive either radiotherapy plus icotinib or radiotherapy treatment.

8.1.3 Sample Size

The number of patients required was estimated according to the methodology by Lakatos and Edward for clinical trials [15]. We assume the 2-year overall survival rate can increase from 30% to 50% with the addition of icotinib to concurrent radiotherapy for esophageal cancer [16]. With a minimum of 2-year follow-up and considering a 5% missing data, the trial needs a recruitment of 127 patients (63 in the control group and 64 in the combination treatment group) to have a power of 80% to detect an improvement in 2-year survival rate. Approximately 127 subjects will be enrolled into this study.

8.1.4 Statistical methods

All the statistical analyses were performed using SPSS version 20.0 software (SPSS Inc., Chicago, IL, USA). Chi-square test was used to evaluate differences in patient characteristics and treatment toxicities in the two groups. Survival analysis were performed by using the Kaplan–Meier method and compared with the log-rank test. Hazard ratios (HRs) with 95% confidence intervals (CI) were calculated using a Cox regression model. Independent prognostic factors were identified by

multivariate analyses using the Cox proportional hazards model. All statistical analyses were performed with a two-sided significance value of 0.05.

9. ETHICAL CONSIDERATIONS

9.1. Good Clinical Practice

This study will be conducted in accordance with Good Clinical Practice (GCP), as defined by China Food and Drug Administration (CFDA).

The study will be conducted in compliance with the protocol. The protocol and any amendments and the subject informed consent will receive independent ethics committee of Hangzhou Cancer Hospital, Huaian First People's Hospital, and Lishui Central Hospital approval/favorable opinion prior to initiation of the study.

Personnel involved in conducting this study will be qualified by education, training, and experience to perform their respective tasks.

This study will not use the services of study personnel where sanctions have been invoked or where there has been scientific misconduct or fraud (e.g., loss of medical licensure, debarment).

9.2. Independent Ethics Committee

Before study initiation, the investigator must have written and dated approval/favorable opinion from the independent ethics committee of Hangzhou Cancer Hospital, Huaian First People's Hospital, and Lishui Central Hospital, for the protocol, consent form, subject recruitment materials (e.g., advertisements), and any other written information to be provided to subjects. A copy of the written approval of the protocol and informed consent form must be received by Zhejiang Bata Pharma Inc. before recruitment of subjects into the study.

The investigator or Zhejiang Bata Pharma Inc., Ltd should provide the independent ethics committee with reports, updates and other information (e.g., expedited safety reports, amendments) according to regulatory requirements or institution procedures.

9.3. Informed Consent

Before a subject’s participation in the clinical study, the investigator is responsible for obtaining written informed consent from the subject or legally acceptable representative. Investigators must ensure that subjects are clearly and fully informed about the purpose, anticipated benefits, potential risks regarding the clinical study.

The sample informed consent form will adhere to the ethical principles that have their origin in the Declaration of Helsinki. The original signed informed consent form should be retained in accordance with institutional policy, and a copy of the signed consent form should be provided to the subject or legally acceptable representative.

9.4. Subject Confidentiality

The investigator must ensure that the subject’s confidentiality is maintained. On the CRFs or other documents submitted to Zhejiang Bata Pharma Inc., Ltd, subjects should be identified by their initials, date of birth, and a subject identification number. The consent form must also include a statement that authorized representatives of the company, of the regulatory have direct access to review the subject’s original medical records.

The rights, safety, and well-being of the study subjects are the most important considerations and should prevail over interests of science and society.

10. REFERENCES

1. Mariette C, Piessen G, Triboulet JP: Therapeutic strategies in oesophageal carcinoma: role of surgery and other modalities. Lancet Oncol 8:545-53, 2007

2. Nallapareddy S, Wilding GE, Yang G, et al: Chemoradiation is a tolerable therapy for older adults with esophageal cancer. Anticancer Research 25:3055-3060, 2005

3. Poon RT, Law SY, Chu KM, et al: Esophagectomy for carcinoma of the esophagus in the elderly: results of current surgical management. Ann Surg 227:357-64, 1998

4. Herskovic A, Martz K, al-Sarraf M, et al: Combined chemotherapy and radiotherapy compared with radiotherapy alone in patients with cancer of the esophagus. N Engl J Med 326:1593-8, 1992

5. Minsky BD, Pajak TF, Ginsberg RJ, et al: INT 0123 (Radiation Therapy Oncology Group 94-05) phase III trial of combined-modality therapy for esophageal cancer: high-dose versus standard-dose radiation therapy. J Clin Oncol 20:1167-74, 2002

6. Chino O, Makuuchi H, Machimura T, et al: Treatment of esophageal cancer in patients over 80 years old. Surg Today 27:9-16, 1997

7. Cui Z, Tian Y, He B, et al: Associated factors of radiation pneumonitis induced by precise radiotherapy in 186 elderly patients with esophageal cancer. Int J Clin Exp Med 8:16646-51, 2015

8. Tan F, Shen X, Wang D, et al: Icotinib (BPI-2009H), a novel EGFR tyrosine kinase inhibitor, displays potent efficacy in preclinical studies. Lung Cancer 76:177-82, 2012

9. Pande AU, Iyer RV, Rani A, et al: Epidermal growth factor receptor-directed therapy in esophageal cancer. Oncology 73:281-289, 2008

10. Dragovich T, McCoy S, Fenoglio-Preiser CM, et al: Phase II trial of erlotinib in gastroesophageal junction and gastric adenocarcinomas: SWOG 0127. J Clin Oncol 24:4922-7, 2006

11. Janmaat ML, Gallegos-Ruiz MI, Rodriguez JA, et al: Predictive factors for outcome in a phase II study of gefitinib in second-line treatment of advanced esophageal cancer patients. J Clin Oncol 24:1612-9, 2006

12. Chinnaiyan P, Huang S, Vallabhaneni G, et al: Mechanisms of Enhanced Radiation Response following Epidermal Growth Factor Receptor Signaling Inhibition by Erlotinib (Tarceva). Cancer Research 65:3328-3335, 2005

13. Tortora G, Gelardi T, Ciardiello F, et al: The rationale for the combination of selective EGFR inhibitors with cytotoxic drugs and radiotherapy. Int J Biol Markers 22:S47-52, 2007

14. Rodriguez CP, Adelstein DJ, Rice TW, et al: A phase II study of perioperative concurrent chemotherapy, gefitinib, and hyperfractionated radiation followed by maintenance gefitinib in locoregionally advanced esophagus and gastroesophageal junction cancer. J Thorac Oncol 5:229-35, 2010

15. Iyer R, Chhatrala R, Shefter T, et al: Erlotinib and radiation therapy for elderly patients with esophageal cancer - clinical and correlative results from a prospective multicenter phase 2 trial. Oncology 85:53-8, 2013

16. Zhang XB, Xie CY, Li WF, et al: [Phase II study of radiotherapy plus erlotinib for elder patients with esophageal carcinoma]. Zhonghua Yi Xue Za Zhi 92:1615-7, 2012

17. Zhai Y, Hui Z, Wang J, et al: Concurrent erlotinib and radiotherapy for chemoradiotherapy-intolerant esophageal squamous cell carcinoma patients: results of a pilot study. Diseases of the Esophagus 26:503-509, 2013

18. Xu Y, Zheng Y, Sun X, et al: Concurrent radiotherapy with gefitinib in elderly patients with esophageal squamous cell carcinoma: Preliminary results of a phase II study. Oncotarget 6:38429-39, 2015

19. Hu W, Wang W, Yang P, et al: Phase I study of icotinib, an EGFR tyrosine kinase inhibitor

combined with IMRT in nasopharyngeal carcinoma. Int J Clin Exp Med 8:15675-15683, 2015
