# Supplementary material for: Icotinib With Concurrent Radiotherapy vs Radiotherapy Alone in Older Adults With Unresectable Esophageal Squamous Cell Carcinoma: A Phase II Randomized Clinical Trial
Source: JAMA Netw Open. 2020 Oct 7;3(10):e2019440. doi: 10.1001/jamanetworkopen.2020.19440 (PMC7542309; doi:10.1001/jamanetworkopen.2020.19440)
Supplement: Supplement 2. — eFigure 1. Overall Survival in RT + Icotinib Group eFigure 2. Overall Survival in RT Group eTable 1. Univariate Analysis of Potential Factors Associated With Survival in all ESCC Patients eTable 2. Multivariate Analysis of Prognostic Factors in all ESCC Patients eTable 3. Patterns of First Failure Between Two Groups of Elderly ESCC Patients [file jamanetwopen-e2019440-s002.pdf]

Supplemental Online Content

Luo H, Jiang W, Ma L, et al. Icotinib with concurrent radiotherapy vs radiotherapy alone in older adults with unresectable esophageal squamous cell carcinoma: a phase II randomized clinical trial. *JAMA Netw Open.* 2020;3(10):e2019440. doi:10.1001/jamanetworkopen.2020.19440

eFigure 1. Overall Survival in RT + Icotinib Group
eFigure 2. Overall Survival in RT Group
eTable 1. Univariate Analysis of Potential Factors Associated With Survival in all ESCC Patients
eTable 2. Multivariate Analysis of Prognostic Factors in all ESCC Patients
eTable 3. Patterns of First Failure Between Two Groups of Elderly ESCC Patients

This supplemental material has been provided by the authors to give readers additional information about their work.

© 2020 Luo H et al. *JAMA Network Open.*

eFigure 1. Overall Survival in RT + Icotinib Group

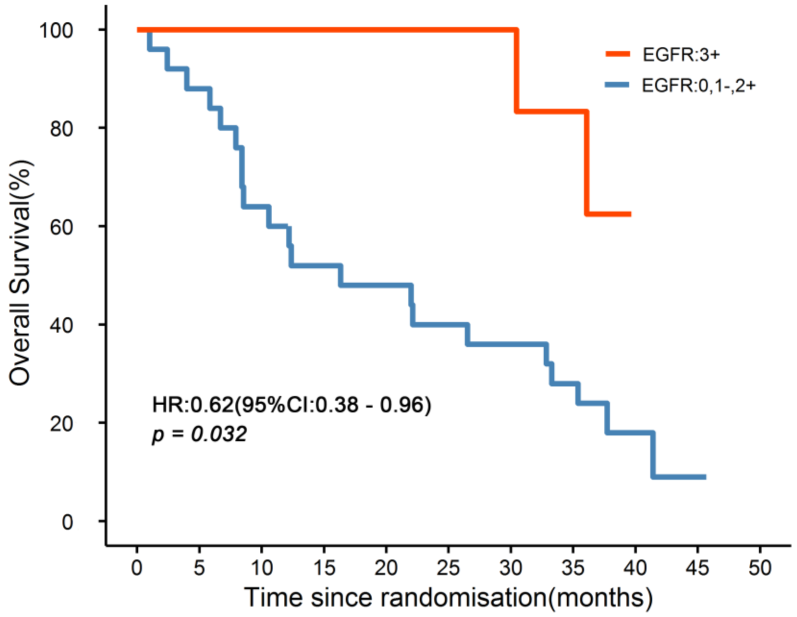

Number at Risk

EGFR:3+	7	6	6	6	6	6	6	5	0	0	0
EGFR:0,1-,2+	25	22	16	13	12	10	9	7	2	1	0

© 2020 Luo H et al. *JAMA Network Open.*

eFigure 2. Overall Survival in RT Group

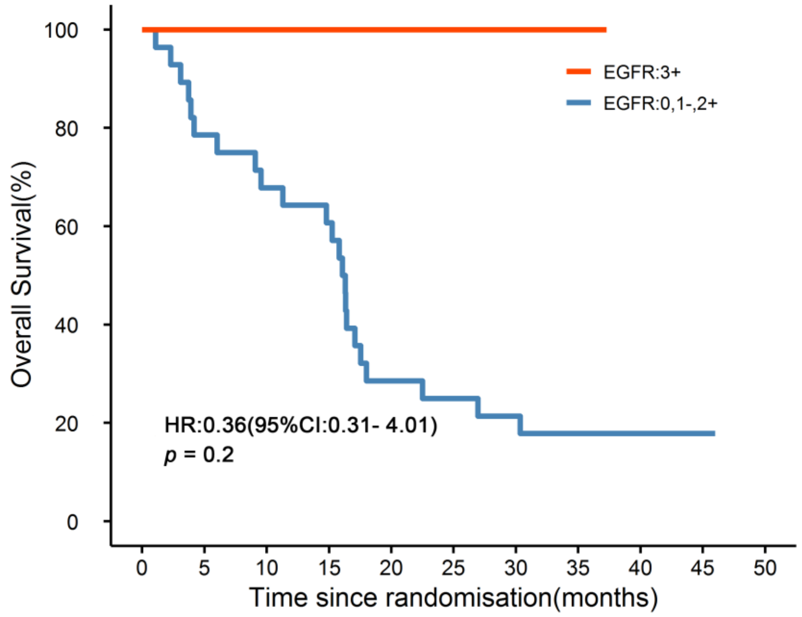

Number at Risk

EGFR:3+	1	1	1	1	1	1	1	1	0	0	0
EGFR:0,1-,2+	28	22	19	17	8	7	6	5	2	1	0

© 2020 Luo H et al. *JAMA Network Open.*

eTable 1. Univariate Analysis of Potential Factors Associated With Survival in all ESCC Patients

Factors	HR	95%CI-low	95%CI-up	p
TKI				
No TKI	reference			
TKI	1.739	1.146	2.638	.009
Sex				
Female	reference			
Male	.663	.430	1.020	.061
Age				
70 ≤ age < 75	reference			
75 ≤ age < 80	.893	.532	1.499	.669
Age ≥ 80	.842	.491	1.444	.532
T stage				
2	reference			
3	.431	.229	.812	.009
4	.582	.347	.977	.040
N stage				
0	reference			
1	.746	.491	1.135	.171
ECOG				
0-1	reference			
2	.780	.518	1.175	.235
Location				
Cervical esophagus	reference			
Upper-third	.488	.184	1.294	.149
Middle-third	.572	.303	1.079	.084
Lower-third	1.074	.648	1.780	.781
Tumor length				
<5	reference			
≥5	.657	.430	1.004	.052
EGFR				
0-2	reference			
3	.427	.15	1.2	.106

Abbreviation: ESCC, esophageal squamous cell carcinoma; HR, hazard ratio; CI: confidence interval; TKI: tyrosine kinase inhibitor; ECOG: Eastern Cooperative Oncology Group performance status.

© 2020 Luo H et al. *JAMA Network Open.*

eTable 2. Multivariate Analysis of Prognostic Factors in all ESCC Patients

Factors	HR	95%CI-low	95%CI-up	p
TKI				
No TKI	Reference			
TKI	1.703	1.090	2.662	.019
Sex				
Female	Reference			
Male	.804	.507	1.274	.353
Age				
70 ≤ age < 75	Reference			
75 ≤ age < 80	1.201	.682	2.117	.526
Age ≥ 80	.962	.554	1.673	.892
T				
2	Reference			
3	.465	.240	.902	.023
4	.644	.378	1.095	.104
N				
0	Reference			
1	.664	.413	1.068	.092
ECOG				
0-1	Reference			
2	.812	.523	1.259	.352
Location				
Cervical esophagus	Reference			
Upper-third	.544	.191	1.555	.256
Middle-third	.542	.268	1.094	.087
Lower-third	1.043	.612	1.779	.876
Tumor length(cm)				
<5	Reference			
≥5	.784	.492	1.249	.306

Abbreviation: ESCC, esophageal squamous cell carcinoma; HR, hazard ratio; CI: confidence interval; TKI: tyrosine kinase inhibitor; ECOG: Eastern Cooperative Oncology Group performance status

© 2020 Luo H et al. *JAMA Network Open.*

eTable 3. Patterns of First Failure Between Two Groups of Elderly ESCC Patients

	RT group (N=63)	RT+icotinib group (N=64)
Local recurrence	36 (57·1%)	24 (37·5%)
Regional recurrence	4 (6·3%)	3 (4·7%)
Distant metastasis	10 (15·9%)	4 (6·3%)
Both local and regional	2 (3·2%)	1 (1·6%)
Both local/regional and distant failure	7 (11·1%)	4 (6·3%)

Values are number (%) unless otherwise stated.

Abbreviation: ESCC, esophageal squamous cell carcinoma; RT, radiation therapy.

© 2020 Luo H et al. *JAMA Network Open.*
