# Supplementary material for: Icotinib With Concurrent Radiotherapy vs Radiotherapy Alone in Older Adults With Unresectable Esophageal Squamous Cell Carcinoma: A Phase II Randomized Clinical Trial
Source: JAMA Netw Open. 2020 Oct 7;3(10):e2019440. doi: 10.1001/jamanetworkopen.2020.19440 (PMC7542309; doi:10.1001/jamanetworkopen.2020.19440)
Supplement: Supplement 3. — Data Sharing Statement [file jamanetwopen-e2019440-s003.pdf]

Data Sharing Statement

Luo. Icotinib With Concurrent Radiotherapy vs Radiotherapy Alone in Older Adults With Unresectable Esophageal Squamous Cell Carcinoma. *JAMA Netw Open*. Published October 07, 2020.
10.1001/jamanetworkopen.2020.19440

Data
Data available: No
